# Communication: Synthesis of a Novel Triphenyltin(IV) Derivative of 2- Mercaptonicotinic Acid with Potent Cytotoxicity *in vitro*

**DOI:** 10.1155/S1565363303000189

**Published:** 2003

**Authors:** Marianna N. Xanthopoulou, Sotiris K. Hadjikakou, Nick Hadjiliadis, Markus Schürmann, Klaus Jurkschat, Jayne Binolis, Spyros Karkabounas, Konstantinos Charalabopoulos

**Affiliations:** 1 Inorganic and Analytical Chemistry Department of Chemistry University of Ioannina Ioannina 45110 Greece; 2 Lehrstuhl für Anorganische Chemie II der Universität Dortmund Dortmund 44221 Germany; 3 Department of Experimental Physiology Medical School University of Ioannina Ioannina 45110 Greece

## Abstract

A novel triphenyltin(IV) derivative of 2-mercaptonicotinic acid (H_2_mna) of formula {[(C_6_H_5_)_3_Sn]_2_(mna).[(CH_3_)_2_CO]} (1) has been synthesized and characterized by elemental analysis and ^1^H, ^13^C-NMR, and FT-IR spectroscopic techniques. The crystal structure of complex (1) has been determined by single crystal X-ray diffraction analysis at 173(1) K. Compound (1) contains two triphenyltin moieties linked by a doubly de-protonated 2,mercaptonicotinic acid (H_>2_mna). It is an example of a pentacoordinated Ph_3_SnXY system with an axial-equatorial arrangement of the phenyl groups at Sn(1). Compound (1), exhibits potent, in vitro, cytotoxicity against sarcoma cancer cells (mesenchymal tissue) from the Wistar rat, polycyclic aromatic hydrocarbons (PAH, benzo[a]pyrene) carcinogenesis.


### COMMUNICATION:

### Synthesis of a Novel Triphenyltin(IV) Derivative of 2-Mercaptonicotinic Acid with Potent Cytotoxicity *in vitro*.

Marianna N. Xanthopoulou[a], Sotiris K. Hadjikakou*[a], Nick Hadjiliadis*[a], Markus Schürmann[b], Klaus Jurkschat[b], Jayne Binolis[c], Spyros Karkabounas[c] and Konstantinos Charalabopoulos[c]

[a] *Inorganic and Analytical Chemistry, Department of Chemistry, University of Ioannina, 45110 Ioannina, Greece.*

[b] *Lehrstuhl für Anorganische Chemie II der Universität Dortmund, 44221 Dortmund, Germany.*

[c] *Department of Experimental Physiology, Medical School, University of Ioannina 45110 Ioannina Greece*

**Abstract**

A novel triphenyltin(IV) derivative of 2-mercaptonicotinic acid ($H_2$mna) of formula {[$(C_6H_5)_3Sn]_2$(mna)·[$(CH_3)_2CO$]} (1) has been synthesized and characterized by elemental analysis and $^1H$, $^{13}C$-NMR, and FT-IR spectroscopic techniques. The crystal structure of complex (1) has been determined by single crystal X-ray diffraction analysis at 173(1) K. Compound (1) contains two triphenyltin moieties linked by a doubly de-protonated 2-mercaptonicotinic acid ($H_2$mna). It is an example of a pentacoordinated $Ph_3SnXY$ system with an axial-equatorial arrangement of the phenyl groups at Sn(1). Compound (1), exhibits potent, in vitro, cytotoxicity against sarcoma cancer cells (mesenchymal tissue) from the Wistar rat, polycyclic aromatic hydrocarbons (PAH, benzo[a]pyrene) carcinogenesis.

**Keywords:** triphenyltin(IV) compound, S ligands-heterocyclic thioamides, mercaptonicotinic acid, crystal structures.

In the course of our work on the synthesis and structure of organotin(IV) complexes derived from heterocyclic thioamides we have studied the reactions of 2-mercaptonicotinic acid ($H_2$mna) (a multi-dentate ligand) (Scheme 1) with triphenyltin chloride. The triphenyltin group was chosen, since it is known to confer biological (fungicidal and acaricidal) properties on the resulting compounds /1/.

---

* All correspondence should be addressed to:

Assistant Professor S.K. Hadjikakou; tel. +30-26510-98374; e-mail: shadjika@cc.uoi.gr

Professor N. Hadjiliadis. +30-26510-98420; fax +30-26510-44831; e-mail: nhadjil@cc.uoi.gr, tel

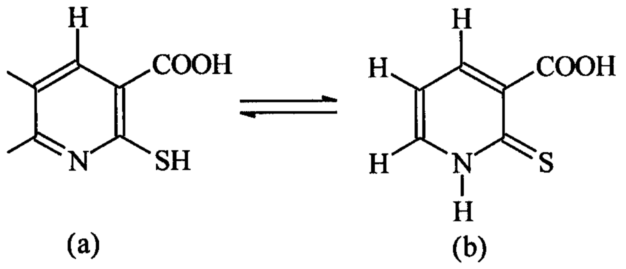

**Scheme 1.** Thiole-thione tautomerism of $H_2$mna.

In this paper we report the synthesis and characterization of a novel triphenyltin(IV) complex with the heterocyclic thioamide 2-mercaptonicotinic acid ($H_2$mna) of formula $\{[(C_6H_5)_3Sn]_2(mna)\cdot[(CH_3)_2CO]\}$ (**1**). Crystal structure of complex (**1**) has been determined by X-ray diffraction at 173(1) K.

Complex (**1**) has been synthesized by reacting a methanolic solution of triphenyltin chloride $Ph_3SnCl$ with an aqueous solution of 2-mercapto-nicotinic acid ($H_2$mna) which contains a twofold amount of potassium hydroxide. The structure was solved by direct methods SHELXS97 /2a/ and successive difference Fourier syntheses. Refinement applied full-matrix least-squares methods SHELXL97 /2b/. Atomic scattering factors for neutral atoms and real and imaginary dispersion terms were taken from International Tables for X–ray Crystallography /2c/. Intensity data for the colourless crystals were collected on a Nonius KappaCCD diffractometer with graphite-monochromated MoKα radiation at 173 K. $C_{45}H_{39}NO_3SSn_2$, MW = 911.21, monoclinic in $P2_1/n$, a = 9.1148(2), b = 29.2819(6), c = 15.5556(4)Å, β = 106.2851(9)°, V = 3985.19(16) $Å^3$, Z = 4, Dx = 1.519 $Mg/m^3$, F(000) = 1824, λ(MoKα) = 0.71073 Å, μ = 1.346 $mm^{-1}$, T = 173(1) K, final R = 0.028 for 5797 unique observed [F> 4.0σ(F)] diffractometer data. Measurements of *in vitro* cells toxicity have been carried out in preliminary repetitions according to the method described in literature /3/.

The ir spectrum of complex (**1**) shows distinct absorptions at 1556 and 1330 $cm^{-1}$, which are assigned to ν(CN) vibrations (thioamide I and II bands) and at 1073 and 656 $cm^{-1}$, which are attributed to the ν(CS) vibrations (thioamide III and IV bands). The corresponding thioamide I and II bands in crystalline $H_2$mna (**2**) appear at 1562 and 1310 $cm^{-1}$ while thioamide bands III and IV are observed at 1071 and 650 $cm^{-1}$ /4/.

A diagram of compound (1) as well as selected bond lengths and angles are shown in Figure 1.

The structure of compound (1) consists of two [$Ph_3$Sn(IV)] moieties bridged by a doubly de-protonated 2-mercaptonicotinic acid ($H_2$mna) molecule. The Sn(1) atom exhibits a distorted trigonal bipyramidal configuration with C(11), C(31), and S(1) occupying the equatorial and N(1) and C(21) occupying the axial positions. The distortion from the ideal trigonal bipyramidal geometry is manifested (i) in the N(1)-Sn(1)-C(21) angle of 158.72(7)° being the result of ligand constraint (four-membered Sn(1)-N(1)-C(1)-S(1) ring), (ii) the displacement by 0.5181 Å of Sn(1) from the plane defined by C(11), C(31) and S(1) (plane A), and (iii) by the geometrical goodness /5/ ΔΣ(θ) = 29.3° reflecting the position along the path tetrahedron(0°)→trigonal bipyramid(90°). The latter indicates the tin atom in compound **1** to be almost midway between both configurations. This is (i) in line with the Sn(1)-N(1) bond distance of 2.711(2) Å being 0.561 Å longer than the covalent radii of nitrogen (0.75 Å) /6/ and tin (1.40 Å) /6/ and (ii) represents a straightforward example for an intramolecular nucleophilic face-attack at a tetrahedral tin atom. As a consequence, the Sn(1)-C(21) bond distance (the one becoming axial) of 2.160(2) Å is slightly longer than

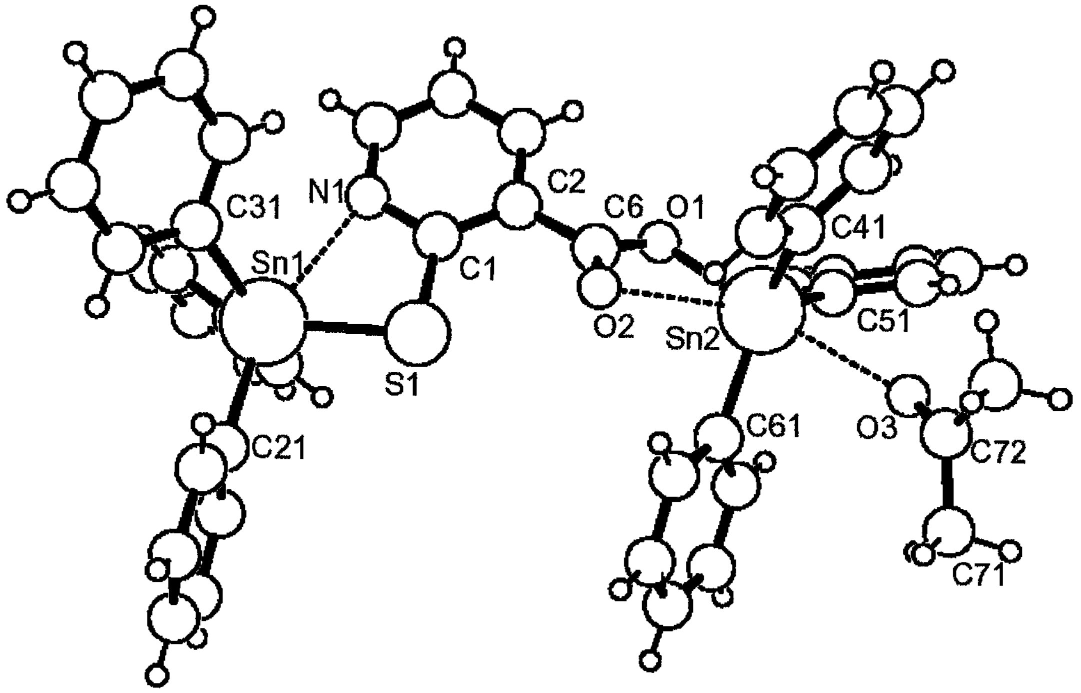

**Fig. 1:** ORTEP diagram of compound (**1**) together with the atomic numbering scheme. Selected bond lengths (Å) and angles [°]; Sn(1)-S(1) = 2.4450(7), Sn(1)-N(1) = 2.711(2), S(1)-C(1) = 1.759(2), Sn(1)-C(11) = 2.121(2), Sn(1)-C(31) = 2.140(2), Sn(1)-C(21) = 2.160(2), Sn(2)-O(1) = 2.1149(15), Sn(2)-O(2) = 2.7779(16), Sn(2)-O(3) = 2.7763(18), Sn(2)-C(61) = 2.121(2), Sn(2)-C(51) = 2.137(2), C(11)-Sn(1)-C(31) = 114.83(9), C(11)-Sn(1)-C(21) = 106.69(9), C(31)-Sn(1)-C(21) = 107.52(10), C(61)-Sn(2)-C(41) = 127.20(10), C(61)-Sn(2)-C(51) = 109.63(9), C(41)-Sn(2)-C(51) = 114.21(10).

the Sn(1)-C(11) and Sn(1)-C(31) distances (the ones becoming equatorial) of 2.121(2) and 2.140(2) Å, respectively. The Sn(1)-S(1) bond distance of 2.4450(7) Å is as expected [$(C_6H_{10})_3$Sn(mbzt)] (mbzt = 2-mercapto-benzothiazole) /7/ (Sn-S = 2.472), [$(CH_3)_3Sn(PhN_2C_2S_3)$] ($PhN_2C_2S_3$ = 5-mercapto-3-phenyl-1,3,4-thiadiazoline-2-thione) /8/ (Sn-S = 2.5409(9) Å) and in [$(C_6H_5)_3$Sn(pmt)] (pmtH = 2-mercapto-pyrimidine) /9/ (Sn-S = 2.442(3) Å).

The Sn(2) atom also exhibits a distorted trigonal bipyramidal configuration (geometrical goodness /5/ $\Delta\Sigma(\theta)$ = 52.44°) with C(41), C(51), and C(61) occupying the equatorial and O(1) and O(3) occupying the axial positions. The Sn(2) atom is displaced by 0.3683 Å in direction of O(1) from the equatorial plane being defined by C(41), C(51) and C(61) (plane **B**). The O(1)-Sn(2)-O(3) angle amounts to 176.23(6)° and the angle between planes A and B is 74.96°. Moreover, according to Reedijk's geometric parameter ($\tau$ =($\beta$-$\alpha$)/60) /10/ the calculated $\tau$ values are 0.73 and 0.82 for Sn(1) and Sn(2) respectively, being equal to zero for perfectly tetragonal pyramidal geometry and unity for perfectly trigonal pyramidal /10/.

The carboxylate group coordinates Sn(2) in an anisobidentate mode with Sn(2)-O(1) = 2.1149(15) and Sn(2)-O(2) = 2.7779(16) Å. The acetone molecule is weakly coordinated to tin, with Sn(2)-O(3) = 2.7763(18) Å.

Compound (**1**) was tested for anti-tumor potential against sarcoma cells (mesenchymal tissue) from the Wistar rat, polycyclic aromatic hydrocarbons (PAH, benzo[a]pyrene) carcinogenesis. Cytotoxic activity for complex (**1**) was evaluated as % percentage of the cell survived in variable concentrations (1 to 0.025 mM) of the complex after 24 hours. Table 1 summarizes cytotoxic activity of complex **1** and the corresponding activity of *cis*- Platin:

**Table 1.**

Cytotoxic activity for complex (1) and *cis*-Platin in variable concentrations (1 to 0.025 mM) of the complexes after 24 hours.

| **C (mM)** | *control* | 0.025 | 0.050 | 0.075 | 0.100 | 0.250 | 0.500 | 0.750 | 1.000 | **Ref** |
|---|---|---|---|---|---|---|---|---|---|---|
| **Complex (1)** | 100 | 0.00 | 0.00 | 0.27 | 0.00 | 0.00 | 0.00 | 0.00 | 0.00 | * |
| ***cis*-Platin** | 100 | 14.70 | 11.90 | - | 6.25 | 3.10 | 2.70 | 10.90 | 1.70 | /11/ |

* This work.

These results show strong antiproliferative effects of (**1**) in the concentration range explored.

### ACKNOWLEDGEMENTS.

This work was carried out in partial fulfilment of the requirements for a M.Sc. thesis of Ms Marianna N. Xanthopoulou within the research programme financed by the research committee of the University of Ioannina-Greece and coordinated by Assistant Professor S.K. Hadjikakou. The graduate program EPEAEK in Bioinorganic Chemistry financed by the Ministry of Education of Greece and coordinated by Professor N. Hadjiliadis is also acknowledged for the instrumental support.

### REFERENCES

1. A.G. Davies and P.J. Smith, in G. Wilkinson (Ed.), *Comprehensive Organometalhc Chemistry*, Pergamon Press, New York, 1982.
2. [a] G.M. Sheldrick; Programs for Crystal Structure Analysis (Release 97-2). Institut fur Anorganische Chemie der Universität, Tammanstrasse 4, D-3400 Göttingen, Germany, (1998). [b] *International Tables for Crystallography*, 1992. Vol. C Dordrecht: Kluwer Academic Publishers. [c] G.M. Sheldrick, 1997. SHELXTL. Release 5.1 Software Reference Manual, Bruker AXS, Inc., Madison, Wisconsin, USA.
3. M.N. Xanthopoulou, S.K. Hadjikakou, N. Hadjiliadis, M. Schürmann, K. Jurkschat, A. Michaelides, S. Skoulika, T. Bakas, J. Binolis, S. Karkabounas and K. Charalabopoulos, *submitted for publication*.

4. P.C. Zachariadis, S.K. Hadjikakou , N. Hadjiliadis Adonis Michaelides, S. Skoulika, Y. Ming and Y. Xiaolin, *Inorg. Chim. Acta*, **343**, 361-365 (2003).
5. U. Kolb, M. Beuter and M. Draeger, *Inorg. Chem.*, **33**, 4522-4530 (1994).
6. J. E. Huheey, E. A. Keiter and R. L. Keiter; *Inorganic Chemistry-Principles of Structure and Reactivity,* 4th ed.; Harper Collins College Publishers, New York, 1993.
7. K.C. Molloy, T.G. Purcell, D. Cunningham, P. McCardle and T. Higgins, *Appl. Organomet. Chem.*, **1**, 119-131 (1987).
8. V. Berceanc, C. Crainic, I. Haiduc, M.F. Mahon, K.C. Molloy, M.M. Venter and P.J. Wilson, *J. Chem. Soc., Dalton Trans.*, **2002**, 1036–1045.
9. L. Petrilli, F. Caruso and E. Rivarola, *Main Group. Metal Chemistry,* **17**, 439-446 (1994).
10. A.W. Addison, T. Nageswara Rao, J. Reedijk, J. Van Rijn and G.C. Verschoor, *J. Chem. Soc. Dalton Trans.*, 1349-1356 (1984).
11. R. Liasko, S. Karkabounas et.al, *unpublished results.*

